# Correction: Camptothecin suppresses NRF2–ARE activity and sensitises hepatocellular carcinoma cells to anticancer drugs

**DOI:** 10.1038/s41416-019-0527-1

**Published:** 2019-07-26

**Authors:** Feng Chen, Huihui Wang, Jiayu Zhu, Rui Zhao, Peng Xue, Qiang Zhang, M Bud Nelson, Weidong Qu, Bo Feng, Jingbo Pi

**Affiliations:** 1 Program of Environmental Toxicology, School of Public Health, China Medical University, No. 77 Puhe Road, Shenyang North New Area, Shenyang, 110122 China; 2 Department of Interventional Radiology, The First Affiliated Hospital of China Medical University, No. 155 Nanjing North Road, Heping Area, Shenyang, 110001 China; 3 Interventional Department, Qianfoshan Hospital, Shandong University, No. 16766 Jingshi Road, Jinan, 250014 China; 4 School of Forensic Medicine, China Medical University, No. 77 Puhe Road, Shenyang North New Area, Shenyang, 110122 China; 5 Key Laboratory of Public Health Safety of Ministry of Education, School of Public Health, Fudan University, P.O. Box 249, 138 Yi Xue Yuan Road, Shanghai, 200032 China; 6 Department of Environmental Health, Rollins School of Public Health, Emory University, Atlanta, GA 30322 USA; 7 MedBlue Incubator, Inc., Research Triangle Park, NC 27709 USA

**Correction to**: *British Journal of Cancer* (2017) **117**, 1495–1506; 10.1038/bjc.2017.317; published online 14 September 2017

The original version of this article contained an error in Fig. 6A. The volumes of the tumour xenografts were incorrectly calculated. The correct figure and figure legend are below, where the volume has been calculated using V = length × width^2^ × π/6. The interpretation of the data and conclusions are not affected.

**Fig. 6:**
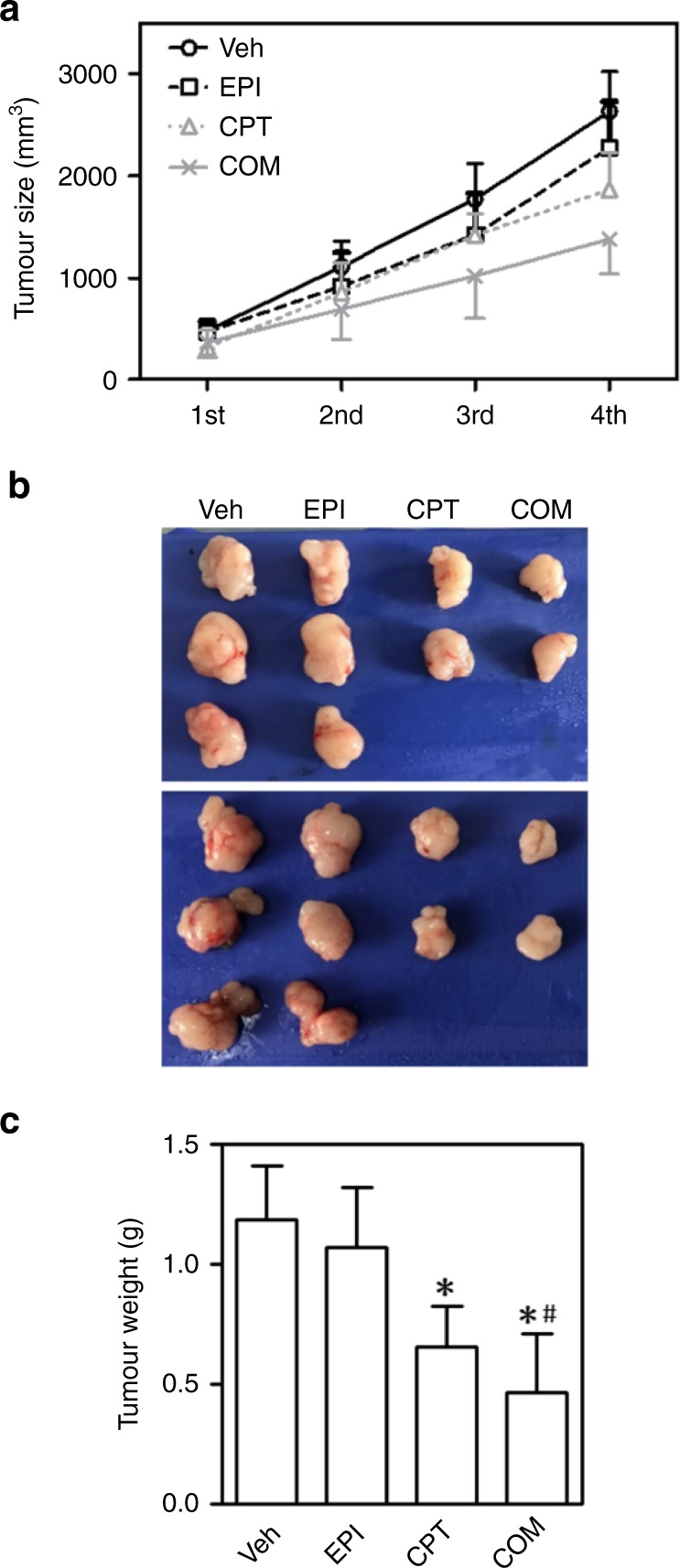
CPT sensitized HCC xenografts to EPI treatment. Nude mice were injected with SMMC-7721 cells. CPT, Camptothecin; EPI, epirubicin. Mice were treated with CPT, EPI or in combination (COM) twice a week for a total of three times. At the end of the experiments, tumours were excised and weighed at the end of the experiment. **a** Tumour growth curves for SMMC-7721 xenografts. **b** Representative images of excised tumours. **c** Excised xenograft tumours weights at the end of the experiment. *n* = 8–10; *p < 0.05 vs. Veh control. ^#^*p* < 0.05 vs EPI

